# Quantum Control of Resonance Lifetimes in Molecular Photodissociation with Intense Laser Fields

**DOI:** 10.1021/acs.jctc.4c01677

**Published:** 2025-02-10

**Authors:** Alberto García-Vela

**Affiliations:** Instituto de Física Fundamental, Consejo Superior de Investigaciones Científicas, Serrano, 123, Madrid 28006, Spain

## Abstract

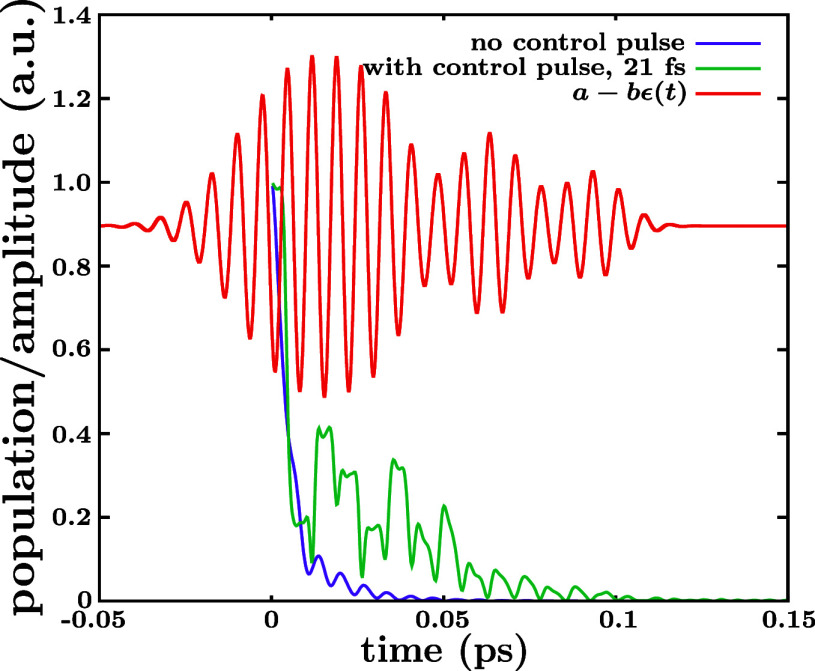

Control of molecular reaction dynamics has been pursued in the last decades. Among these reactions are molecular photodissociation processes governed by resonances. Controlling the lifetime of such resonances imply to control the time duration of the processes. Here, some control schemes that apply moderately intense laser fields are proposed to modify (reducing or increasing) a resonance lifetime. The control strategy applies an intense field as a way to generate a new effective coupling that produces a resonance decay different from the natural one, with a different decay lifetime. In particular, different control schemes are suggested to reduce the lifetime of a long-lived resonance, and to increase the lifetime of a short-lived resonance. A large degree and flexibility of control both in the reduction and in the increase of the resonance lifetime is demonstrated. The experimental applicability of the schemes is discussed. The present schemes thus open the possibility of extensive and universal control of molecular photodissociation processes mediated by resonances.

## Introduction

Quantum control of molecular reaction dynamics has raised an increasing interest over the last decades.^1−34^ The initial control schemes designed by Tannor-Rice-Kosloff^3,4^ and Brumer and Shapiro^2,5,6^ were later followed by more general optimal control methods.^7,8,18^ Control of molecular processes takes advantage of the coherent properties of light. The advent of ultrashort laser pulses in the femtosecond^35−37^ and attosecond^21,25,26,38,39^ time scales made possible an unprecedented time resolution in the control experiments. The development of intense laser fields in the last years also meant a remarkable advance in the control area. Several control schemes applying both weak and strong laser fields have been proposed for different molecular reactions. The weak-field control schemes are based on exploiting quantum interference between different reaction pathways, while strong-field ones rely on modifying the molecular Hamiltonian. Nowadays, molecular reaction control is exerted by applying weak or strong ultrashort laser pulses, combining pulse shaping techniques with learning algorithms.^9,10,14−19^

A large variety of molecular processes are governed by the decay of resonance states. Among them are photodissociation processes like electronic,^40^ vibrational,^41,42^ and rotational^43^ predissociation of a molecular system, as well as low-temperature reactive^44−50^ and nonreactive^51−58^ molecular collisions. The control of resonance-mediated processes is closely related to the control of the underlying resonance decay process, whose behavior is determined by the resonance properties. One of these properties is the resonance lifetime, which determines the duration of the resonance-mediated molecular process. Thus, modifying the corresponding resonance lifetime provides an effective means of control over the resonance-mediated process of interest. More specifically, one can define the modification of a resonance lifetime as the achievement of either a shorter or longer lifetime than the natural one associated with the resonance in the absence of control. Since the resonance decay is modified, the most important implication of the new lifetime is that at a given time the population of the molecular system involved is now different from that found in the natural resonance decay. Such a control of the time duration of a molecular process can be determinant in cases where that process is an intermediate step within a more complex chemical cycle of reactions. Indeed, modifying the duration of a resonance-mediated intermediate process, which implies changing the survival time of the corresponding intermediate chemical species, will alter the subsequent molecular processes of the cycle. In this sense, control of the intermediate process becomes actually equivalent to the control of the final outcome of the chemical cycle.

Control of a resonance lifetime has been pursued in the last years by applying weak fields in the regime of both overlapping^24,59,60^ and isolated^61^ resonances. The control schemes used typically relied on quantum interference induced between resonances by the application of two delayed weak laser pulses. Large increases of the resonance lifetime were achieved. In the present work the control of a resonance lifetime is approached from a different perspective. Instead of exploiting the quantum interference produced by weak laser pulses, moderately intense fields are now applied with the purpose of modifying the intensity of the couplings responsible for the resonance decay. It is shown that the intense fields applied can vary the intensity of these couplings in the direction of increasing or reducing it, thus correspondingly reducing or increasing the resonance lifetime. To the best of the author’s knowledge, this is the first time that intense external fields are used to modify the lifetime of a resonance state. The performance of the control schemes suggested is illustrated by applying them to a model of the photodissociation of the CH_3_ radical. This system is very convenient for the present purpose, because it features different types of resonances, including long-lived and short-lived ones, which can be used to show how the control schemes can reduce or increase, respectively, their decay lifetime.

## Theoretical Background

**Figure 1:**
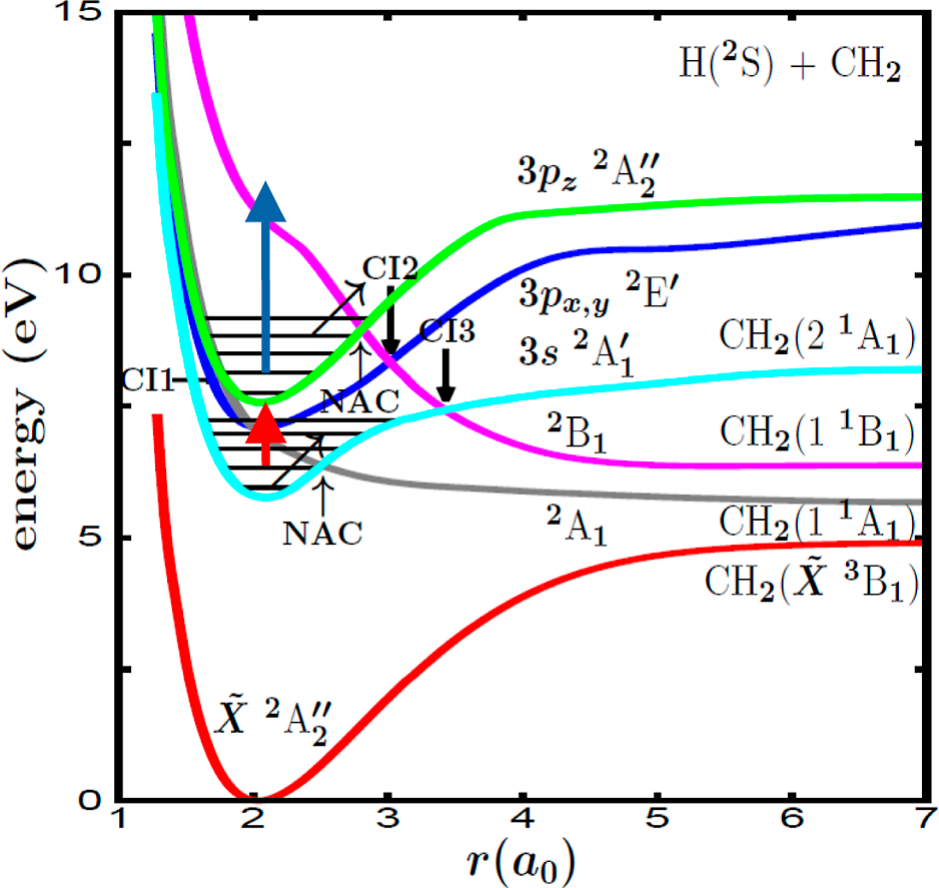
Potential-energy curves of CH_3_ along the CH_2_–H coordinate are shown for the ground electronic state (*X̃*^2^A_2_^″^) and for the first excited states, including the 3s ^2^A_1_^′^, 3p_*x*,*y*_^2^E′, and 3p_*z*_^2^A_2_^″^ Rydberg states and the ^2^A_1_ and ^2^B_1_ valence states. In the figure the position of maximum intensity of the nonadiabatic couplings (NAC) leading to predissociation from the 3s and 3p_*z*_ Rydberg states are indicated by vertical arrows pointing upward. The position of the three conical intersections between 3p_*z*_ and ^2^A_1_ (CI1), between 3p_*x*,*y*_ and ^2^B_1_ (CI2), and between 3s and ^2^B_1_ (CI3) are also marked by horizontal or vertical arrows pointing downward. Two arrows tilted to the right indicate the passage from 3s to ^2^A_1_ and from 3p_*z*_ to ^2^B_1_ through predissociation. The first energy levels of the CH_3_ (*v*, 3s) and CH_3_ (*v*, 3p_*z*_) resonances for *v* = 0–4 are shown in the corresponding potential-energy curves. The two color arrows indicate the excitations with intense fields of the CH_3_ (*v* = 1, 3s) (red arrow) and CH_3_ (*v* = 1, 3p_*z*_) (blue arrow) resonances whose decay is modified in this work.

The photodissociation dynamics of the methyl radical is described by representing the system in the dissociative coordinate along the fragmentation pathway CH_3_ → CH_2_ + H. The nonadiabatic fragmentation dynamics of methyl is simulated by means of a one-dimensional (1D) wave packet treatment applied in the diabatic representation of the different electronic states involved in the photodissociation process. Such a treatment includes the first five excited electronic states of methyl, namely the three 3s, 3p_*x*,*y*_, and 3p_*z*_ Rydberg states and the two dissociative ^2^A_1_ and ^2^B_1_ valence states, as well as all the nonadiabatic couplings between them. The PECs and the nonadiabatic couplings associated with the above excited states were calculated by means of a high-level ab initio internally contracted multireference configuration interaction (MRCI) method.^62^ The PECs are displayed in Figure 1.

The 1D nonadiabatic wave packet model was previously applied in order to investigate the CH_3_ predissociation dynamics in the 3s and 3p_*z*_ Rydberg states.^63^ For simplicity, in that work the dynamics started from the corresponding initial resonance in either the 3s or the 3p_*z*_ state. The excitation of the 3s or 3p_*z*_ resonance (in principle 3p_*x*,*y*_ is a dark state) from the ground electronic state by means of a pump laser pulse was not considered in the wave packet model, assuming that resonance excitation had occurred previously. In the present simulations, and also for simplicity, the same criterion is used and the resonance excitation from the ground electronic state is again not included in the dynamical model used here. Including the resonance excitation from the ground electronic state, would just convolute the resonance population decay with the envelope function of the pump laser pulse used for the excitation. That is, if an isolated resonance, whose population decay is exponential, is excited with a Gaussian laser pulse, the final shape of the time dependence of the resonance population decay will be a convolution of the Gaussian and exponential shapes of the pulse used and of the natural resonance decay. The effect of this convolution with the pump pulse shape makes somewhat less clear the visualization of the control effects on the resonance population decay. A detailed description of the wave packet dynamical treatment applied here, and of the calculation of the resonance wave functions has been given elsewhere.^63^ In the following this description is briefly reviewed, emphasizing the new features of the treatment, like the application of intense fields coupling excited electronic states.

In this work two specific resonances of CH_3_ have been chosen in order to illustrate the performance of the control schemes suggested to modify the resonance lifetime. These resonances are the *v* = 1 resonances of the 3p_*z*_ and 3s Rydberg states, where *v* is the quantum number labeling the resonance energy position, and thus *v* = 1 means the first excited resonance supported by each electronic state. The reason to choose the CH_3_ (*v* = 1, 3p_*z*_) and CH_3_ (*v* = 1, 3s) resonances is because they are a long-lived and a very short-lived resonance, respectively, and therefore they are ideal candidates to apply control schemes to reduce or to increase their lifetime. The decay dynamics of each of these resonance states are simulated by solving the time-dependent Schrödinger equation
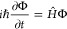
1where the wave packet Φ is expressed as
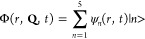
2where **Q** denotes the electronic coordinates, |*n* > denotes the five electronic states 3p_*z*_ (*n* = 1), ^2^B_1_ (*n* = 2), 3p_*x*,*y*_ (*n* = 3), 3s (*n* = 4), and ^2^A_1_ (*n* = 5), *r* is the dissociative CH_2_–H coordinate, and ψ_*n*_(*r*, *t*) are the nuclear wave packets generated in the different electronic states during the photodissociation dynamics.

The time evolution of Φ(*r*, **Q**, *t*) is determined by the evolution of the nuclear packets ψ_*n*_(*r*, *t*), which is governed by eq 1. A difference with the wave packet model previously applied,^63^ is that now a radiation–matter interaction term that couples by means of an intense field the 3p_*z*_ and 3s Rydberg states with the ^2^B_1_ and ^2^A_1_ valence states, respectively, is added to the molecular Hamiltonian. Thus, for the CH_3_ (*v* = 1, 3p_*z*_) resonance eq 1 becomes in matrix form eq 3, while for the CH_3_ (*v* = 1, 3s) resonance eq 1 becomes eq 4.
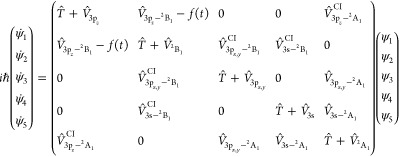
3
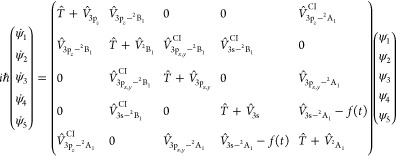
4

In eqs 3 and 4, *T̂* is the kinetic energy operator of *Ĥ*, , with  being the reduced mass associated with *r*; the potential terms in the diagonal of the Hamiltonian matrix (, , ...) are the potential-energy operators associated with the different electronic states; the nonzero off-diagonal terms correspond to the nonadiabatic couplings between the electronic states, with , , and  being the couplings leading to electronic predissociation from the three Rydberg states, and , , and  being the couplings associated with the three conical intersections identified previously.^62^ The *f*(*t*) function is the radiation–matter interaction term
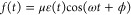
5where μ is the corresponding transition dipole moment (calculated at MRCI ab initio level^62^) coupling radiatively the different Rydberg and valence states, either 3p_*z*_ and ^2^B_1_ or 3s and ^2^A_1_, ε(*t*) is the envelope of the intense laser pulse applied, ω is the excitation photon frequency coupling the resonance of interest in the Rydberg state with the valence state, and ϕ is the pulse phase, which is assumed to be ϕ = 0 for simplicity. In some of the control schemes suggested here ε(*t*) has a Gaussian form
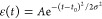
6with σ being the parameter determining the temporal width of the Gaussian pulse (the full width at half-maximum of the pulse, fwhm, is related to σ as fwhm = 2σ(2 ln 2)^1/2^ ≈ 2.355σ), while in other schemes the laser pulses used are more complex. The parameters of the fields applied in the different control schemes are given in next section, where the results are presented and discussed. In all cases (with and without control), the resonance survival probability along time is calculated as the projection
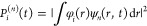
7where φ_*i*_(*r*) is the resonance wave function (associated with the CH_3_ (*v* = 1, 3p_*z*_) and CH_3_ (*v* = 1, 3s) resonances), and ψ_*n*_(*r*, *t*) is the nuclear wave packet corresponding to the 3p_*z*_ and 3s Rydberg states.

By adding in eqs 3 and 4 the *f*(*t*) radiation–matter interaction to the  and  natural couplings leading to predissociation in the 3p_*z*_ and 3s Rydberg states, respectively, with an intense enough ε(*t*) field, new effective couplings  and  are generated. Such new couplings feature a different nature and intensity than the natural ones, leading to a resonance decay governed by a different lifetime. Since the strategy of the control schemes is to modify the natural predissociation couplings, the radiative coupling *f*(*t*) must also connect the two electronic states coupled by predissociation. It is noted that the present control strategy is closely related to the control schemes that rely on generating light-induced potentials (LIPs) and light-induced conical intersections (LICIs).^13,23,27,32,64,65^

## Results and Discussion

As described in the previous section, the present simulations of the resonance decay by electronic predissociation in CH_3_ apply a 1D wave packet model involving several nonadiabatically coupled electronic states. The main reason to apply a 1D reduced-dimensionality model to describe the CH_3_ photodissociation dynamics, is because a complete ab initio representation of all the excited electronic PECs and all the nonadiabatic couplings between them involved in the process is available only in 1D.^62^ It is emphasized, however, that the qualitative behavior of a resonance state and of the coupling leading to the resonance decay is independent of the dimensionality of the dynamical model used, which supports the validity of the main results and conclusions reported here.

### Reduction of the Lifetime of a Long-Lived Resonance by Intense-Field Control Using a Single Gaussian Pulse

**Figure 2:**
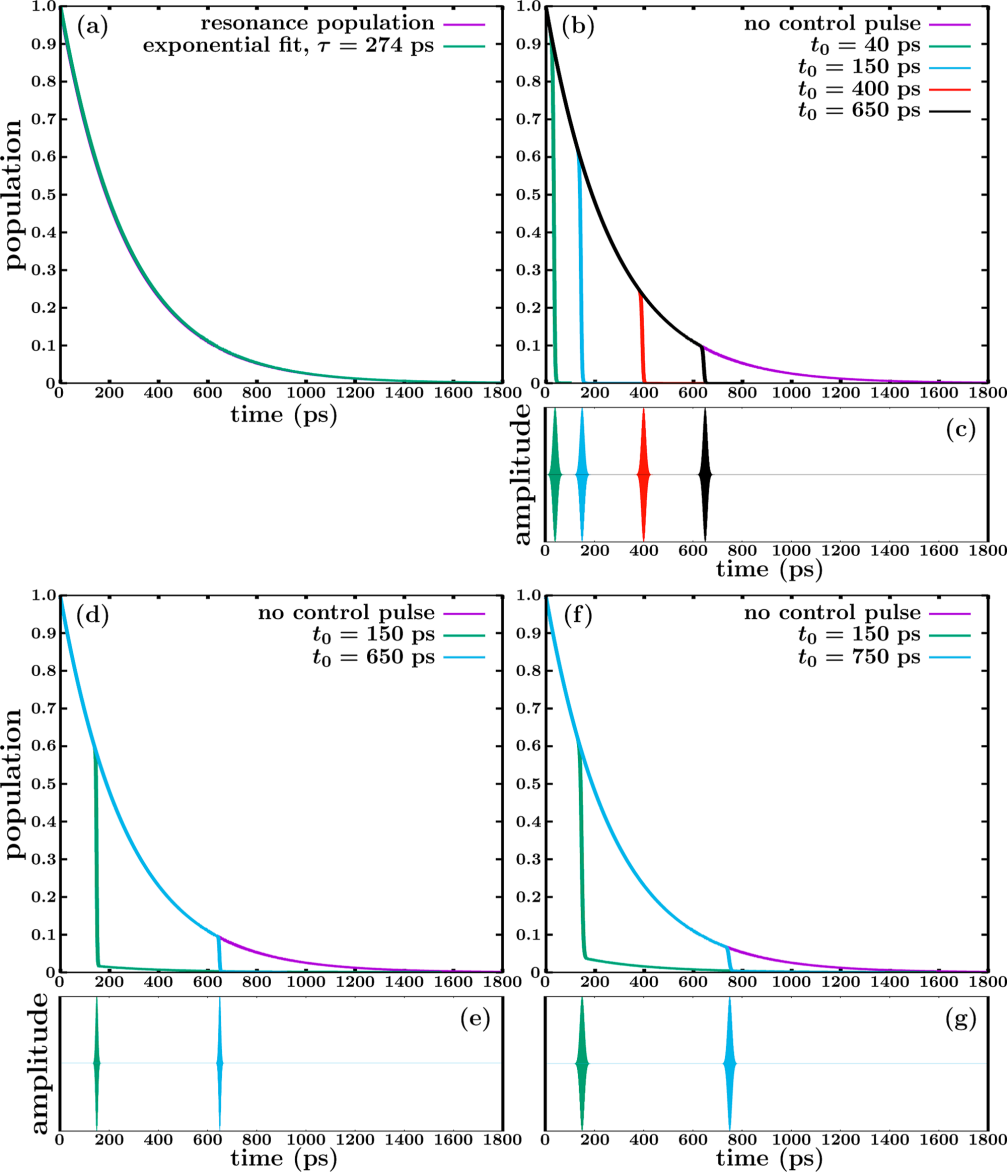
(a) Decay of the CH_3_ (*v* = 1, 3p_*z*_) resonance by predissociation to the ^2^B_1_ valence state in the absence of control, and fit of the population curve to an exponential function e^–*t*/τ^ from which the lifetime τ = 274 ps is extracted. (b) Resonance population decay obtained in the absence of control, and superimposed are shown the population decays calculated after applying four moderately intense Gaussian pulses at different times to excite the CH_3_ (*v* = 1, 3p_*z*_) resonance to the ^2^B_1_ valence state. (c) The four Gaussian pulses applied in (b) with the center times *t*_0_ = 40, 150, 400, and 650 ps (see eq 6). All the pulses have a peak intensity of *I* = 5.3 × 10^11^ W/cm^2^ and a fwhm of 20 ps. (d) Resonance population decay obtained in the absence of control, and superimposed are shown the population decays calculated after applying two moderately intense Gaussian pulses at different times to excite the CH_3_ (*v* = 1, 3p_*z*_) resonance to the ^2^B_1_ valence state. (e) The two Gaussian pulses applied in (d) with the center times *t*_0_ = 150, and 650 ps, with a peak intensity of *I* = 5.3 × 10^11^ W/cm^2^ and a fwhm of 10 ps. (f) Resonance population decay obtained in the absence of control, and superimposed are shown the population decays calculated after applying two moderately intense Gaussian pulses at different times to excite the CH_3_ (*v* = 1, 3p_*z*_) resonance to the ^2^B_1_ valence state. (g) The two Gaussian pulses applied in (f) with the center times *t*_0_ = 150, and 750 ps, with a peak intensity of *I* = 2.0 × 10^11^ W/cm^2^ and a fwhm of 20 ps.

In the previous predissociation dynamics study on CH_3_,^63^ the natural lifetime of the long-lived CH_3_ (*v* = 1, 3p_*z*_) resonance was investigated in the absence of external field, and found to be 274 ps, and its energy position was calculated to be 4579.3 cm^–1^ above the minimum of the 3p_*z*_ PEC. Figure 2a shows the natural population decay of this resonance with time, which is fitted to an exponential function, *e*^–*t*/τ^, from which the resonance lifetime τ = 274 ps is obtained. Since the CH_3_ (*v* = 1, 3p_*z*_) resonance is an isolated one, its decay is exponential and the exponential fit is perfectly superimposed to the population decay curve. Figure 2a shows that the resonance population takes about 1800 ps to decay completely to zero.

Control over the CH_3_ (*v* = 1, 3p_*z*_) resonance lifetime is now exerted by applying a moderately intense Gaussian pulse at different times along the resonance decay, to excite the resonance to the ^2^B_1_ valence state. An excitation energy *E* = ℏω = 27,000 cm^–1^ is used, where ω is the photon frequency of the pulse (see eq 5). The pulses applied have a peak intensity of *I* = 5.3 × 10^11^ W/cm^2^ and a fwhm of 20 ps. The pulses are applied at four different times, determined by their center times at *t*_0_ = 40, 150, 400, and 650 ps (see eq 6) The new population decay curves obtained after application of each pulse are displayed in Figure 2b, and the Gaussian pulses used are shown in Figure 2c.

Figure 2b shows that each pulse is able to quench completely the resonance population during the time it operates. The quenching of the resonance population is the result of modifying the natural coupling  (see eq 3) leading to the resonance decay by predissociation of Figure 2a, by adding the radiation–matter interaction term *f*(*t*) = με(*t*) cos(ω*t* + ϕ), where ε(*t*) cos(ω*t* + ϕ) are the Gaussian pulses displayed in Figure 2c, as discussed in the previous section (see eqs 3, 5, and 6). When the term *f*(*t*) is intense enough, it causes the effect of increasing the intensity of the new, effective coupling , that now is able to produce the complete decay to zero of the resonance population during the operation of the laser pulse. The only difference with the natural decay of the resonance in the absence of control, is that now the fraction of the resonance population excited to the ^2^B_1_ state by the control pulse with a photon energy *E* = 27,000 cm^–1^ will produce fragments in ^2^B_1_ with a larger energy content (with an energy excess of 27,000 cm^–1^) than those produced at the resonance energy before the control pulse is applied. An interesting feature of this control scheme is that by choosing the time at which the pulse is applied (determined by the pulse time center *t*_0_), it is possible to control completely the duration of the resonance decay. Indeed, Figure 2b shows that the resonance population can be completely quenched at short times like 40 or 150 ps, or at longer times like 400 or 650 ps. This feature provides a large degree of flexibility on the control achieved over the resonance lifetime. It is also noted that the scheme is very simple to implement, since it only requires a moderately intense pulse (with a Gaussian or other shape) with a temporal width of a few ps, which can be generated experimentally in a routine way nowadays.

It is now interesting to discuss the conditions under which the present control scheme works. The essential condition to achieve complete quenching of the resonance population is that the modified effective coupling  reaches enough intensity for that purpose. Since the transition dipole moment, μ, does not change, such intensity can only be provided by the laser field ε(*t*) cos(ω*t* + ϕ) during the time that it operates. There are two parameters that determine the ability of the pulse to quench the resonance population, and therefore to reduce its lifetime. One of them is clearly the peak intensity, *I*, and the other one is the temporal width of the pulse, which determines the pulse duration. By increasing these two parameters enhances the effectiveness of the pulse to quench the resonance population, so a clever balance between them helps to obtain the best performance of the control scheme with the minimum cost in terms of pulse energy.

Figure 2b shows that complete quenching of the population can be achieved with the same pulse, regardless the amount of population to be quenched (the shorter the time when the pulse is applied, the larger the amount of population to be quenched). However, what is expected is that the minimum peak intensity and temporal width of the pulse required will depend on the specific amount of population to be quenched. This is illustrated in Figure 2d–g. In Figure 2d two pulses (shown in Figure 2e) are applied at 150 and 650 ps, both with a peak intensity of *I* = 5.3 × 10^11^ W/cm^2^ and a fwhm of 10 ps. Thus, these pulses have the same peak intensity but half the duration than the pulses of Figure 2c. The pulse applied at 650 ps, when the surviving population is already rather small, is able to quench it completely. However, the pulse applied at a shorter time, 150 ps, is not able to quench completely the much larger amount of resonance population still present at that time, due to the shorter duration of the pulse with respect to that used at the same time in Figure 2b. Once the pulse is over, the amount of population still not quenched will follow the natural resonance decay, since now only the  predissociation coupling is acting.

Similarly, in Figure 2f two pulses (shown in Figure 2g) are applied at 150 and 750 ps, both with a peak intensity of *I* = 2.0 × 10^11^ W/cm^2^ and a fwhm of 20 ps. In this case the pulses have the same temporal width as the pulses of Figure 2c, but less than half the peak intensity of those. And also similarly, the pulse applied at the long time of 750 ps can quench completely the small population still remaining, while the pulse applied at 150 ps is again unable to quench all the population surviving at that time, due to its lower intensity compared to the corresponding pulse used in Figure 2b. Thus, the basic condition to achieve complete decay of the resonance population at a given time, is to find the right combination of peak intensity and temporal width of the pulse that ensures enough intensity and duration to quench all the resonance population at the chosen time.

### Reduction of the Lifetime of a Long-Lived Resonance by Intense-Field Control Using a Train of Gaussian Pulses

**Figure 3:**
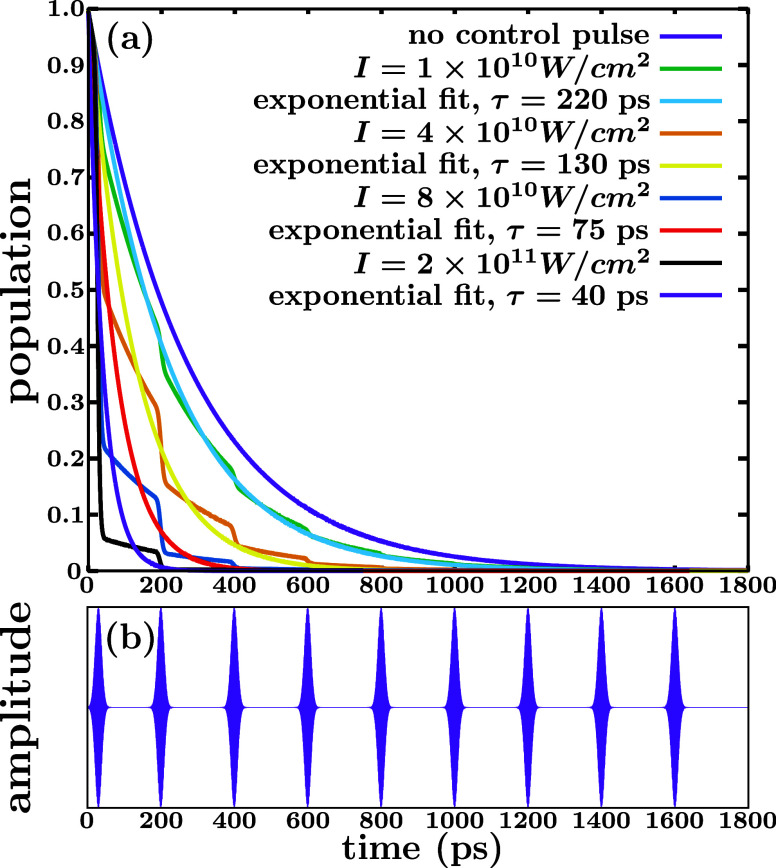
(a) Decay of the CH_3_ (*v* = 1, 3p_*z*_) resonance by predissociation to the ^2^B_1_ valence state in the absence of control, and population decays obtained after applying four trains of Gaussian pulses with increasing peak intensities to excite the CH_3_ (*v* = 1, 3p_*z*_) resonance to the ^2^B_1_ valence state. Each resonance population decay curve has been fitted to an exponential function e^–*t*/τ^, which is shown, and the lifetime τ obtained from the fit is indicated in the figure. (b) The train of Gaussian pulses applied, where the only parameter that varies for the different trains is the peak intensity *I* of all the pulses of the train, also indicated in panel (a). The fwhm of the pulses is 20 ps.

The control scheme described above is among the simplest schemes one can think of to reduce a resonance lifetime. A somewhat more complicated scheme can be designed by applying a train of pulses, which in the current application have a Gaussian shape. Figure 3a displays the results obtained by applying such a pulse train (shown in Figure 3b), when the peak intensity of all the Gaussian pulses is increased. The same excitation energy as before, *E* = 27,000 cm^–1^, is used. Trains with four different peak intensities of all the pulses (indicated in Figure 3a) are applied, and in all cases the pulses have a fwhm of 20 ps and a separation between them of 200 ps. Similarly to the results of Figure 2, the effect of each pulse of the train on the resonance population is a partial decrease or quench of this population during the time that the pulse is operating. The combined effect of all the pulses of the train produces an overall decrease of the resonance population in a stepwise way. The population curves can be viewed as a “stairway of population decreases”. As the pulse peak intensity increases, the decrease of the population caused by each pulse becomes more pronounced, which in turn also produces a larger overall decrease of the resonance population and therefore of the lifetime. The four population curves obtained with the different pulse trains have been fitted to exponential functions in order to estimate the associated lifetimes. It is found that the natural lifetime of 274 ps can be reduced to 220, 130, 75, and 40 ps with the peak intensities used, which are not high, of the order of 10^10^ to 10^11^ W/cm^2^.

The control scheme using a pulse train provides an alternative strategy to that based on a single Gaussian pulse. Instead of causing a sudden quench of the resonance population (as shown in Figure 2), it produces a more gradual decrease of the population. This allows for a larger degree of control of the resonance population at all times, while with a single Gaussian pulse the resonance decay at times before the pulse is applied cannot be controlled. An advantage of the single-pulse scheme is that the pulse energy is always constant, since the only parameter varied to control the duration of the resonance survival is the time center of the pulse. In the pulse-train scheme the energy of the train increases when the intensity of the Gaussian pulses increases. It is noted, however, that this energy increase is actually smaller than it might appear. The reason is that as the intensity of the pulses of the train increases, the resonance decay time decreases, and only part of the train is actually required to achieve complete disappearance of the population. For instance, for the pulse intensity *I* = 4 × 10^10^ W/cm^2^ the resonance population has decayed to zero after 600 ps, and therefore the train pulses for *t* > 600 ps are no longer required. A train consisting only of four Gaussian pulses up to 600 ps would be enough in this case. Thus, the increase of energy of the pulse train scales actually more slowly than linearly with the factor of increase of the pulse intensity.

**Figure 4:**
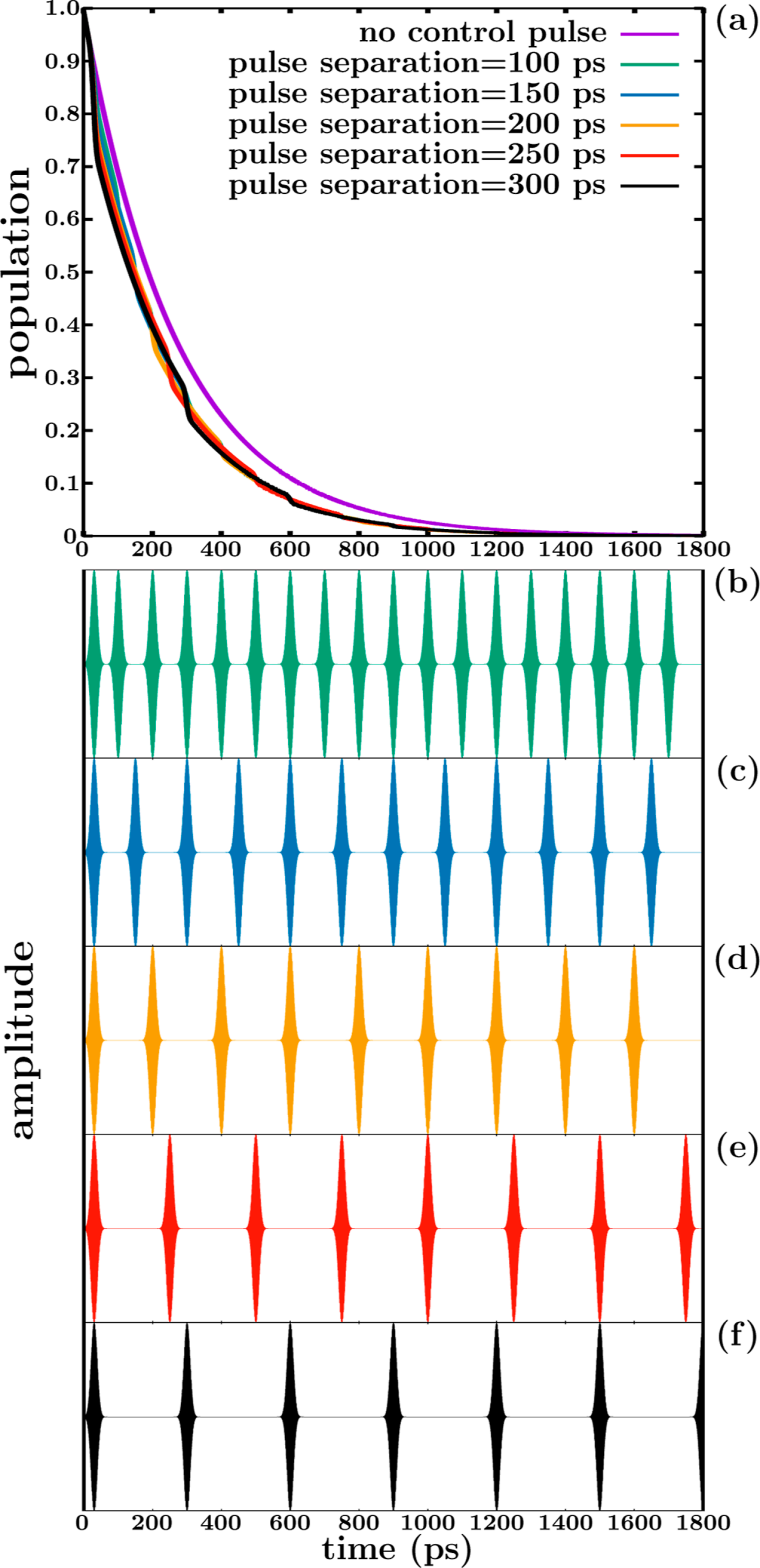
(a) Decay of the CH_3_ (*v* = 1, 3p_*z*_) resonance by predissociation to the ^2^B_1_ valence state in the absence of control, and population decays obtained after applying five trains of Gaussian pulses with a decreasing repetition rate to excite the CH_3_ (*v* = 1, 3p_*z*_) resonance to the ^2^B_1_ valence state. (b–f) The trains of Gaussian pulses with a decreasing repetition rate applied in (a). In all the trains, all the pulses have a fwhm of 20 ps and a peak intensity of *I* = *c* × 10^10^ W/cm^2^, where the factor *c* is adjusted for each train such that the area associated with the square of all the pulse trains shown in (b–f) is the same.

In the results of Figure 3 control over the resonance lifetime by applying a pulse train is achieved by increasing the pulse intensity. An interesting question is whether it is still possible to induce control effects over the resonance population by applying different pulse trains with a constant energy all of them. This possibility is investigated by using pulse trains with different repetition rates, that is, with different time separations between the Gaussian pulses of the train. Five trains are applied with a decreasing repetition rate, and the calculated resonance decay curves are shown in Figure 4a, while the five trains used are displayed in Figures 4b–f. Again, the excitation energy *E* = 27,000 cm^–1^ is used. In the case of the train with a time separation between pulses of 200 ps (Figure 4d), the parameters of the pulse are *I* = 1.0 × 10^10^ W/cm^2^ and a fwhm of 20 ps. The population curve obtained with this pulse is the same as that of Figure 3 associated with the lifetime τ = 220 ps. The pulses of the other four trains have the same temporal width, and intensities *I* = *c* × 10^10^ W/cm^2^, where *c* is a factor that is adjusted for each train such that the area associated with the square of all the pulse trains of Figure 4b–f becomes the same constant value. Thus, all the trains applied have the same energy.

The population curves obtained with the five pulse trains display the same overall resonance decay, with the same associated lifetime τ = 220 ps. This is not surprising, since now all the trains have the same energy, and thus the intensity of the effective coupling  is globally the same for the different trains. However, the specific shape of the five population curves along time is different. The origin of this different shape is that the partial decreases of the population produced by each Gaussian pulse of the train occur at different times for the different trains. While for long times (*t* > 600 ps) all the curves display a very similar shape, for relatively short times (*t* < 300 ps) the differences in shape between the curves become more pronounced. As a result, at short times there can be differences of 20%–30% of population between the different curves. Such differences can be important in the cases where the occurrence of further steps in a chemical cycle depends strongly on the amount of resonance population present at a given time. Thus, even keeping constant the energy of the different pulse trains, still significant control effects can be achieved by varying the pulse repetition rate.

### Increase of the Lifetime of a Short-Lived Resonance by Intense-Field Control

**Figure 5:**
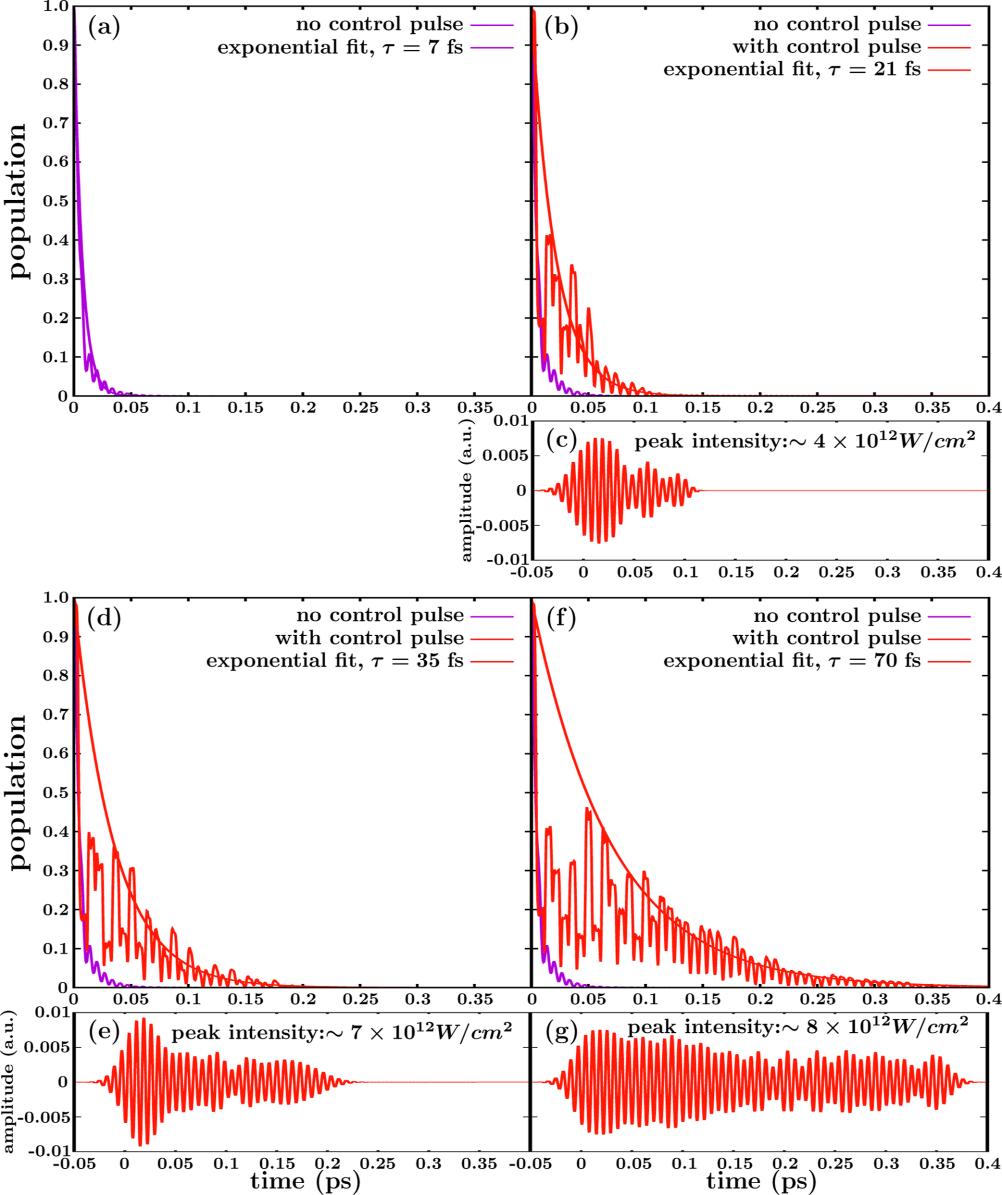
(a) Decay of the CH_3_ (*v* = 1, 3s) resonance by predissociation to the ^2^A_1_ valence state in the absence of control, and fit of the population curve to an exponential function e^–*t*/τ^ from which the lifetime τ = 7 fs is extracted. (b) Population decay obtained after applying the pulse shown in (c), with a peak intensity of *I* ∼ 4 × 10^12^ W/cm^2^, to excite the CH_3_ (*v* = 1, 3s) resonance to the ^2^A_1_ valence state. The population curve has been fitted to an exponential function e^–*t*/τ^, which is shown, and the lifetime τ obtained from the fit is indicated in the figure. (d) Same as in (b) after applying the pulse shown in (e). (f) Same as in (b) after applying the pulse shown in (g). See the text for details.

The lifetime of the short-lived CH_3_ (*v* = 1, 3s) resonance was calculated to be 7 fs, and its energy position was found to be 4604.8 cm^–1^ above the minimum of the 3s PEC.^63^Figure 5a shows the population decay of this resonance with time, along with a fit to an exponential function, e^–*t*/τ^, from which the resonance lifetime is obtained. Despite the isolated CH_3_ (*v* = 1, 3p_*z*_) resonance, the CH_3_ (*v* = 1, 3s) one is a broad resonance that overlaps to some extent with other resonances in the 3s Rydberg state. This overlap is the origin of the small undulations shown by the resonance population curve of Figure 5a. Such undulations, however, are irrelevant for the present control purpose.

Control over the lifetime of the CH_3_ (*v* = 1, 3s) resonance is achieved by applying three different pulses to excite the resonance to the ^2^A_1_ valence state with an excitation energy *E* = ℏω = 4400 cm^–1^. The three pulses used are more complex than the previous Gaussian pulses or trains of Gaussian pulses applied to reduce the resonance lifetime. Such more complex pulses are built up “by hand”, by adding a series of short and frequently overlapping in time Gaussian pulses. In the following, these nonGaussian single pulses shown in Figure 5 will be denoted by ϵ(*t*). In Figure 5b–g the new resonance decay curves obtained along with the pulses applied are displayed. The pulses have associated an increasing peak intensity of the order of 10^12^ W/cm^2^ in all cases, as well as an increasing time duration.

The curves of Figure 5b,d,f (calculated with eq 7) show that by applying these three pulses the resonance survival is indeed increased remarkably, depending on the time duration of each specific pulse used. By fitting the longer-living population curves obtained to exponential functions, the associated modified lifetimes can be estimated. With the pulses used the resonance natural lifetime of 7 fs is increased up to 21 (by a factor of 3), 35 (by a factor of 5), and 70 fs (by a factor of 10). Further lifetime enhancements could be achieved by using longer pulses. The energy of the three pulses of Figure 5c,e,g is estimated by calculating the integral *g* = ∫|ϵ(*t*)|^2^ d*t*, and the three values found are *g* = 0.056, 0.09, and 0.175 au, respectively. Thus, interestingly the ratio of increase of the energy of the pulses is somewhat smaller than the ratio of increase of the resonance lifetime, despite that the shape of the pulses is not currently optimized.

**Figure 6:**
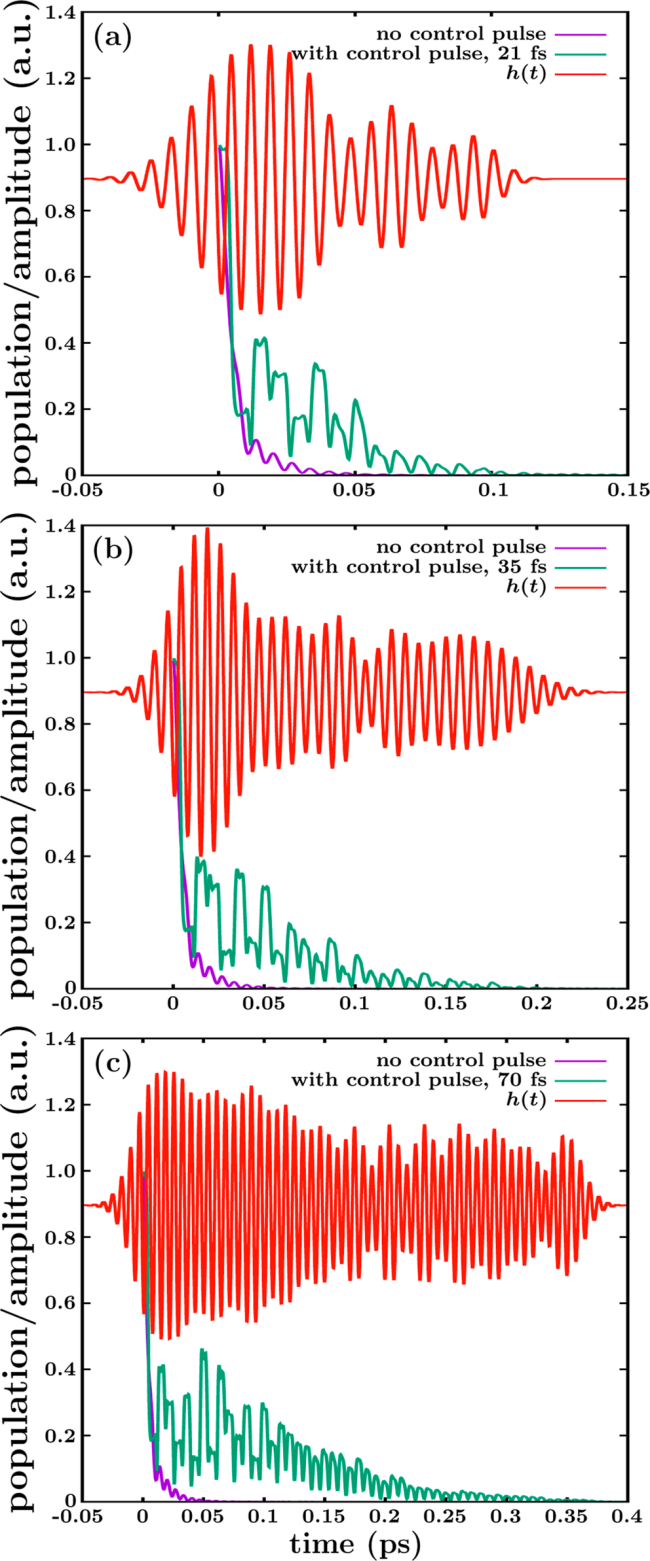
(a) Decay of the CH_3_ (*v* = 1, 3s) resonance by predissociation to the ^2^A_1_ valence state in the absence of control, population decay obtained after applying the pulse shown in Figure 5c, and the magnitude *h*(*t*) associated with this pulse and calculated with eq 8. (b) Same as in (a) by applying the pulse shown in Figure 5e. Same as in (a) by applying the pulse shown in Figure 5g.

The shape of all the longer-living decay curves display a structure of peaks with a certain degree of overlap. Such a structure of peaks is apparently related with the structure of oscillations in time of the three different pulses of Figure 5c,e,g applied. In order to check this possibility, the magnitude *h*(*t*), defined as
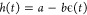
8has been calculated for the three pulses used in Figure 5. In eq 8, *a* and *b* are constants defined as  and *b* = ∫μ(*r*) d*r*. Thus, *h*(*t*) is essentially a simplified form of the new effective  coupling [being now *f*(*t*) = μϵ(*t*)], where for simplicity the *r*-coordinate dependence of  and μ has been removed by integration to leave only the time dependence in *h*(*t*), in order to compare with the time-dependent decay curves of Figure 5b,d,f. This comparison is shown in Figure 6.

The magnitude *h*(*t*) shows a structure of undulations that is the same structure of temporal oscillations of the field ϵ(*t*) applied (multiplied by *b*). When the pulse is off, *h*(*t*) = *a* ∼ 0.9 au The interesting finding is that the maxima of the peaks of the three resonance decay curves coincide with the minima of the oscillations of the corresponding *h*(*t*) curves, and vice versa. Since *h*(*t*) provides a measure of the intensity of the new effective  coupling, the implication of this result is that indeed, when the intensity of the effective coupling decreases [leading to a decrease of *h*(*t*)] the resonance survival probability increases, and vice versa. Thus, the structure of peaks of the decay curves of Figure 5 is actually produced by and follows the structure of oscillations of the ϵ(*t*) pulses applied. This implies that the intensity of the resonance population peaks can be controlled with the intensity of the pulse oscillations. This is why the smaller-amplitude oscillations of ϵ(*t*) [or *h*(*t*)] at longer times produce peaks of decaying intensity at those times in the population curves. For the same reason, the enhancement of time duration of the resonance survival can also be controlled by varying the duration of the ϵ(*t*) pulse.

**Figure 7:**
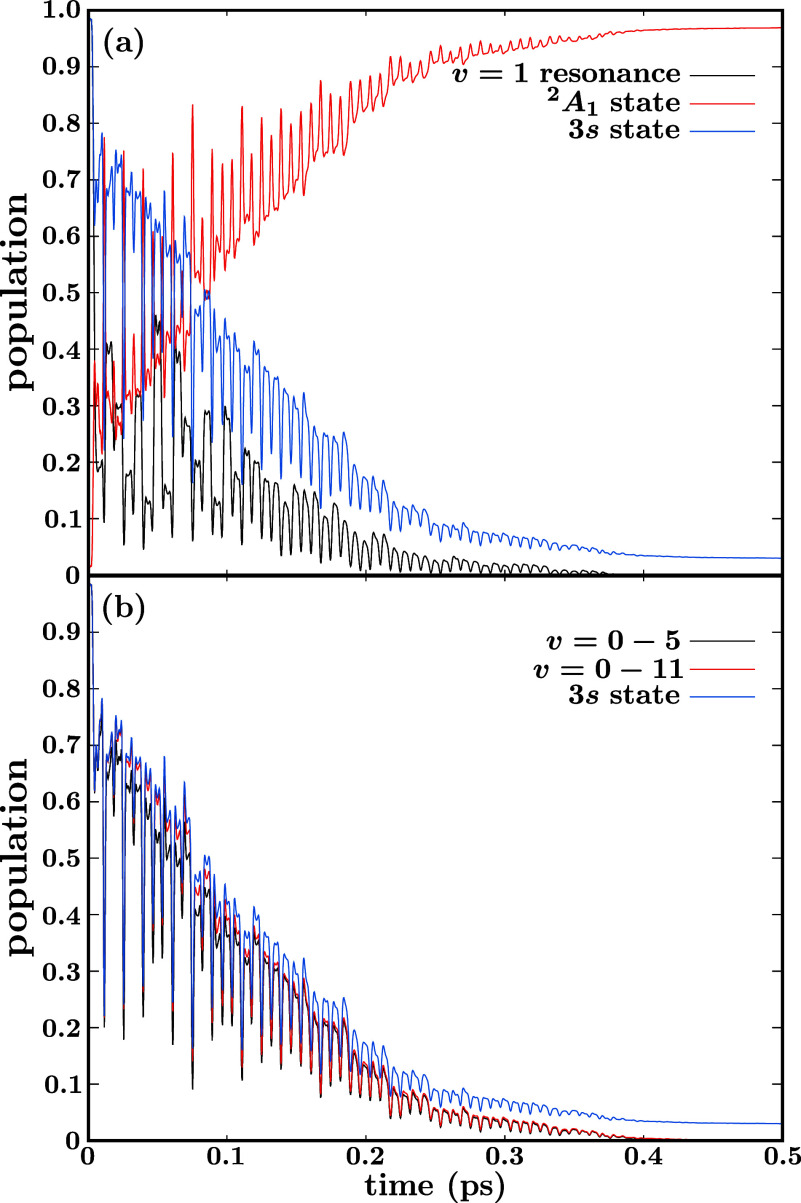
(a) CH_3_ (*v* = 1, 3s) resonance population decay curve calculated by applying the pulse of Figure 5g to excite the resonance to the ^2^A_1_ valence state (black curve, the same decay curve as in Figure 5f). Also shown are the total time-dependent populations in the ^2^A_1_ valence state (red curve) and in the 3s Rydberg state (blue curve). (b) Sum of the time-dependent populations corresponding to the first six resonances (*v* = 0–5, black curve) and to the first 12 resonances (*v* = 0–11, red curve) in the 3s state, and total population in the 3s state (blue curve, the same curve as in Figure 7a).

It is now interesting to analyze the detailed mechanism by which the ϵ(*t*) field drives the temporal enhancement of the CH_3_ (*v* = 1, 3s) resonance survival. To this purpose, several time-dependent populations which are produced upon the resonance decay under the influence of the control pulse are analyzed. For this analysis, the control pulse chosen was that shown in Figure 5g, and the corresponding time-dependent populations are shown in Figure 7. Figure 7a displays the CH_3_ (*v* = 1, 3s) resonance decay curve obtained under the control pulse effect (the same curve as that of Figure 5f), and the total populations in the states ^2^A_1_ and 3s [calculated as ⟨ψ_5_(*r*,*t*)|ψ_5_(*r*,*t*)⟩ and ⟨ψ_4_(*r*,*t*)|ψ_4_(*r*,*t*)⟩, respectively; see eq 2].

The curves of Figure 7a show several interesting findings. As expected, the population in the ^2^A_1_ valence state increases with time up to nearly 1, since all the resonance population finally decays to this state. The sum of the total populations in the ^2^A_1_ and 3s states is always 1 along time. More surprising in principle is the result that the population in 3s is remarkably larger (particularly at short times) than the CH_3_ (*v* = 1, 3s) resonance population. This indicates that other resonances (*v* = 0 and *v* > 1; the 3s state supports 14 vibrational resonances, *v* = 0–13) are being populated from the very beginning of the CH_3_ (*v* = 1, 3s) resonance decay. This is confirmed by the curves of Figure 7b which, in addition to the total population in the 3s state, display the sum of populations in the first six resonances (*v* = 0–5) and in the first 12 resonances (*v* = 0–11). The population in each resonance is calculated with eq 7. The curves show that by increasing the number of resonances included in the sum of populations, the total amount of population in the 3s state is gradually approached. Therefore, the mechanism induced by the control pulse to increase the duration of the CH_3_ (*v* = 1, 3s) resonance survival is also causing the population of the remaining resonances of 3s. Another implication of this result is the following. Since the sum of the populations in the ^2^A_1_ and 3s state is 1 along time, the velocity of their increase and decrease, respectively, is closely connected and determined by the specific pulse applied. Indeed, as the time duration of the laser pulse applied increases, the velocity of decrease (increase) of the 3s (^2^A_1_) population will be reduced. This means that, although the resonance population will decay finally to the continuum, the time it takes to complete that decay is also determined by the duration of the pulse applied.

The most interesting result of the curves of Figure 7 is that all of them feature the same structure of peaks, which is the structure shown by the resonance decay curve of Figure 5f. As discussed above, this structure is closely related to and reflects the temporal oscillations of the pulse ϵ(*t*) applied. The time separation between the maxima (or minima) of the peaks in the structures of all the curves of Figure 7 is ∼7 fs, which is the same separation between the maxima (or minima) of the oscillations of the ϵ(*t*) pulse of Figure 5g. The presence of the same structure of maxima and minima in all the curves of Figure 7 implies that the control pulse is inducing along time an exchange of population between the CH_3_ (*v* = 1, 3s) resonance and the ^2^A_1_ state, but also between the ^2^A_1_ state and all the resonances of the 3s state. This exchange of population is confirmed by the result that, while both the CH_3_ (*v* = 1, 3s) resonance and the 3s total population curve display a structure of peaks in phase (with coinciding maxima and minima), the phase of the structure of the ^2^A_1_ curve is the opposite (the maxima of the ^2^A_1_ curve coincide with the minima of the other two curves, and vice versa).

The postulated mechanism which is consistent with the present results is the following. Initially only the CH_3_ (*v* = 1, 3s) resonance is populated. When the control pulse begins to operate it pumps resonance amplitude to the ^2^A_1_ state (at an excitation energy of 4400 cm^–1^ above the resonance energy), and this amplitude starts rolling down the slope of the repulsive potential curve of ^2^A_1_ (see Figure 1). Since the control pulse is intense enough, it also operates in the opposite sense, transferring part of the amplitude pumped to the ^2^A_1_ state back to the CH_3_ (*v* = 1, 3s) resonance, since the excitation photon energy is the same. The temporal sequence would be that half a temporal oscillation of ϵ(*t*) (which takes ∼7 fs) would excite resonance amplitude to ^2^A_1_, producing a peak (and a maximum) in the ^2^A_1_ population, and a minimum in the CH_3_ (*v* = 1, 3s) resonance population curve. Next half oscillation of ϵ(*t*) (for another ∼7 fs) transferring back population from ^2^A_1_ to the resonance, would produce a minimum in the ^2^A_1_ population and a peak (a maximum) in the resonance population curve. This sequence would be repeated for each full ϵ(*t*) oscillation, producing the same structure but with opposite phase in the curves of the resonance and of ^2^A_1_, as found in Figure 7a. This sequence is also consistent with the shape of *h*(*t*) shown in Figure 6.

Now, the additional population of the remaining resonances of 3s with this mechanism is explained as follows. In the effective coupling , both terms  and *f*(*t*) operate coupling 3s and ^2^A_1_ in the two senses, although *f*(*t*) is expected to be the most intense term. The resonance amplitude pumped to ^2^A_1_ by *f*(*t*) constitutes a wave packet that rolls down the potential slope, and when it reaches the region of influence of the predissociation coupling , this coupling transfers back part of the amplitude from ^2^A_1_ to 3s. This amplitude transfer will populate the CH_3_ (*v* = 1, 3s) resonance, but also the other 3s resonances, depending on the overlap between the wave packet moving on ^2^A_1_ and the different wave functions of the 3s resonances. The time scale of this process of amplitude transfer from ^2^A_1_ to 3s through  is expected to be a combination of the time it takes the created wave packet to roll down the potential (expected to be small) and the predissociation lifetime associated with the different resonances (which is also small at least for the lowest 3s resonances^63^). Interestingly, in all the resonance decay curves of Figure 5 the peaks at relatively short times (the biggest peaks) display on top a weak splitting into a structure of a double peak, which is likely to be produced by the action of . Indeed, this weak splitting would reflect a much weaker intensity of  compared to *f*(*t*), as expected, along with a faster time scale of this inverse predissociation process, which is probably occurring in a continuous way from the initial time when resonance amplitude is excited to ^2^A_1_, and is not subject to the temporal ϵ(*t*) oscillations. This process mediated by the  coupling is the likely way to populate 3s resonances different from the CH_3_ (*v* = 1, 3s) one at very short times. But once there is some population in those resonances, ϵ(*t*) will excite them to ^2^A_1_ at different energies (i.e., each resonance energy plus the excitation energy of 4400 cm^–1^), and the same pulse will transfer ^2^A_1_ amplitude back to 3s, populating all the 3s resonances far more efficiently than . In this way the combination of the  and *f*(*t*) couplings along time would populate all the 3s resonances, explaining the population curves of Figure 7. It is noted that the fact that some of the 3s resonances overlap^63^ due to their large energy width, also contributes to the efficient population of the different 3s resonances.

The control scheme mechanism described above acts as “protecting” the resonance population from decay. Indeed, in the absence of control pulse the resonance population decays in a few fs, but when the pulse operates it generates a temporal loop that pumps population from the resonance to the ^2^A_1_ state, and back to the resonance. Thus, the ^2^A_1_ state acts as a “reservoir” where the resonance population is temporarily stored before it returns to the resonance state. Repetition of these cycles of storage and return of the resonance population along the time duration of the control pulse effectively delays the resonance decay, increasing its survival.

In order to investigate the effect of the excitation energy on the control scheme, simulations varying this parameter have been carried out. The vertical excitation energy from the minimum of the 3s state to the potential energy curve of the ^2^A_1_ state is 8661 cm^–1^. Since the CH_3_ (*v* = 1, 3s) resonance is about 4605 cm^–1^ above the 3s minimum, the minimum energy required to excite the resonance to ^2^A_1_ is about 4056 cm^–1^. The energy of 4400 cm^–1^ used in the simulations of Figures 5–7 is only slightly above this minimum energy. Several simulations were performed by increasing the excitation energy above 4400 cm^–1^, using in all cases the laser pulse of Figure 5g. For an excitation energy of 4500 cm^–1^ the results for the enhancement of the resonance survival are very similar to those shown in Figure 5f. The excitation energy was further increased to 4900, 5400, 6400, 7300, 9000, 11,000, and 14,000 cm^–1^. As the energy increases the temporal enhancement of the resonance survival gradually disappears, and the resonance decay curve approaches fast the natural survival curve of Figure 5a. These results thus indicate that the control scheme works best as the excitation energy is as close as possible to the resonance energy, that is, close to the minimum excitation energy required to reach the ^2^A_1_ state. When the excitation energy increases, the resonance population excited to ^2^A_1_ appears to disperse more and more among several excited vibrational states of both ^2^A_1_ and 3s, which causes that bringing back this population to the original resonance state becomes increasingly more difficult.

Before concluding it is interesting to comment on the applicability of the present control schemes. The CH_3_ radical has no special features regarding the application of the suggested control strategies. CH_3_ supports both short-lived and long-lived resonance states, same as many other molecular systems. Since the control schemes have proven to be able to modify the lifetime of both types of resonances, they can be applied to any molecule featuring such resonances. The only requirement is that the molecule must possess a sufficiently high transition dipole moment coupling radiatively the two electronic states connected by the predissociation coupling.

## Conclusions

Different control schemes using moderately intense laser pulses are proposed to modify (reducing or increasing) the lifetime of a resonance state. The strategy underlying is to modify the intensity of the natural coupling leading to the resonance decay with the electric field applied, creating a new effective coupling that produces a different decay of the resonance, with a different lifetime associated. In particular, two control schemes are suggested to reduce the lifetime of a long-lived resonance. One of them applies a single Gaussian-type pulse that is able to quench completely the resonance population during the time it operates. By choosing the specific time at which the pulse is applied, one can achieve the desired reduction of the resonance lifetime. The other scheme applies a train of (Gaussian) pulses. Each pulse of the train causes a partial quench of the resonance population, and all of them combined lead to a global reduction of the resonance survival lifetime. In this case a large degree of control can be exerted by varying the peak intensity of the train pulses. The main condition for the two schemes to work is a combination of enough intensity and duration of the pulses used to achieve complete quenching of the resonance population at the desired time. Due to the rather moderate intensities and temporal widths (a few ps) of the single pulses or pulse trains required, they are simple to implement experimentally with the current technology, and thus its practical application should be straightforward.

Another control scheme is proposed to increase the survival lifetime of a short-lived resonance state. This scheme applies a single moderately intense pulse, albeit with a shape and structure more complex than the Gaussian pulses used in the two previous schemes. The mechanism of this control scheme acts as a sort of “protection” of the resonance population against decay. The pulse induces temporal loops or cycles where the resonance population is temporarily stored in the excited electronic state, and then it returns back to the resonance state. The repetition of these cycles along the pulse duration delays the resonance decay, enhancing its lifetime. Increasing the duration of the pulse allows to increase the resonance survival. In the present work the lifetime is enhanced by a factor of 10, and could be even further increased. It is noted, however, that the resonance lifetime cannot be increased unlimitedly, since fractions of the resonance population are decaying irreversibly all the time, and finally all the population will end up in the continuum. The control scheme suggested only can delay substantially that process. Due to the more complex structure of the laser pulse used in this case, the experimental application of this scheme would require a pulse shaper combined with an adaptive feedback genetic algorithm.^9,10,14−19^ Again, at present this technology is available and well developed. Thus, the schemes proposed here make possible an extensive and universal control of molecular photodissociation processes mediated by resonances.

## References

[ref1] Rice S. A.; Zhao M. Optical Control of Molecular Dynamics; John Wiley and Sons: New York, 2000.

[ref2] Shapiro M.; Brumer P. Quantum Control of Molecular Processes, 2nd ed. Revised and Enlarged; Wiley-VCH: Weinheim, Germany, 2012.

[ref3] Tannor D. J.; Rice S. A. Control of Selectivity of Chemical Reaction via Control of Wave Packet Evolution. J. Chem. Phys. 1985, 83, 5013–5018. 10.1063/1.449767.

[ref4] Tannor D. J.; Kosloff R.; Rice S. A. Coherent Pulse Sequence Induced Control of Selectivity of Reactions: Exact Quantum Mechanical Calculations. J. Chem. Phys. 1986, 85, 5805–5820. 10.1063/1.451542.

[ref5] Brumer P.; Shapiro M. Control of Unimolecular Reactions Using Coherent Light. Chem. Phys. Lett. 1986, 126, 541–546. 10.1016/S0009-2614(86)80171-3.

[ref6] Brumer P.; Shapiro M. Laser Control of Product Quantum State Populations in Unimolecular Reactions. J. Chem. Phys. 1986, 84, 4103–4104. 10.1063/1.450074.

[ref7] Peirce A. P.; Dahleh M. A.; Rabitz H. Optimal Control of Quantum-Mechanical Systems: Existence, Numerical Approximation, and Applications. Phys. Rev. A 1988, 37, 4950–4964. 10.1103/PhysRevA.37.4950. 9899641

[ref8] Kosloff R.; Rice S. A.; Gaspard P.; Tersigni S.; Tannor D. J. Wavepacket Dancing: Achieving Chemical Selectivity by Shaping Light Pulses. Chem. Phys. 1989, 139, 201–220. 10.1016/0301-0104(89)90012-8.

[ref9] Judson R. S.; Rabitz H. Teaching Lasers to Control Molecules. Phys. Rev. Lett. 1992, 68, 1500–1503. 10.1103/PhysRevLett.68.1500. 10045147

[ref10] Assion A.; Baumert T.; Bergt M.; Brixner T.; Kiefer B.; Seyfried V.; Strehle M.; Gerber G. Control of Chemical Reactions by Feedback-Optimized Phase-Shaped Femtosecond Laser Pulses. Science 1998, 282, 919–922. 10.1126/science.282.5390.919. 9794756

[ref11] Anfinrud P.; de Vivie-Riedle R.; Engel V. Ultrafast Detection and Control of Molecular Dynamics. Proc. Natl. Acad. Sci. U.S.A. 1999, 96, 8328–8329. 10.1073/pnas.96.15.8328. 10411871 PMC33622

[ref12] Rabitz H.; de Vivie-Riedle R.; Motzkus M.; Kompa K. Whither the Future of Controlling Quantum Phenomena?. Science 2000, 288, 824–828. 10.1126/science.288.5467.824. 10796997

[ref13] Solá I. R.; Chang B. Y.; Santamaría J.; Malinovsky V. S.; Krause J. L. Selective Excitation of Vibrational States by Shaping of Light-Induced Potentials. Phys. Rev. Lett. 2000, 85, 4241–4244. 10.1103/PhysRevLett.85.4241. 11060608

[ref14] Brixner T.; Damrauer N. H.; Niklaus P.; Gerber G. Photoselective Adaptive Femtosecond Quantum Control in the Liquid Phase. Nature 2001, 414, 57–60. 10.1038/35102037. 11689940

[ref15] Levis R. J.; Menkir G. M.; Rabitz H. Selective Bond Dissociation and Rearrangement with Optimally Tailored, Strong-Field Laser Pulses. Science 2001, 292, 709–713. 10.1126/science.1059133. 11283357

[ref16] Daniel C.; Full J.; González L.; Lupulescu C.; Manz J.; Merli A.; Vajda S.; Wöste L. Deciphering the Reaction Dynamics Underlying Optimal Control Laser Fields. Science 2003, 299, 536–539. 10.1126/science.1078517. 12543966

[ref17] Brixner T.; Gerber G. Quantum Control of Gas-Phase and Liquid- Phase Femtochemistry. ChemPhysChem 2003, 4, 418–438. 10.1002/cphc.200200581. 12785256

[ref18] Vogt G.; Krampert G.; Niklaus P.; Nuernberger P.; Gerber G. Optimal Control of Photoisomerization. Phys. Rev. Lett. 2005, 94, 068305 10.1103/PhysRevLett.94.068305. 15783783

[ref19] Hoki K.; Brumer P. Mechanisms in Adaptive Feedback Control: Photoisomerization in a Liquid. Phys. Rev. Lett. 2005, 95, 168305 10.1103/PhysRevLett.95.168305. 16241849

[ref20] Sussman B. J.; Townsend D.; Ivanov M. I.; Stolow A. Dynamic Stark Control of Photochemical Processes. Science 2006, 314, 278–281. 10.1126/science.1132289. 17038617

[ref21] Znakovskaya I.; von den Hoff P.; Zherebtsov S.; Wirth A.; Herrwerth O.; Vrakking M. J. J.; de Vivie-Riedle R.; Kling M. F. Attosecond Control of Electron Dynamics in Carbon Monoxide. Phys. Rev. Lett. 2009, 103, 103002 10.1103/PhysRevLett.103.103002. 19792301

[ref22] Goto H.; Katsuki H.; Ibrahim H.; Chiba H.; Ohmori K. Strong-Laser-Induced Quantum Interference. Nat. Phys. 2011, 7, 383–385. 10.1038/nphys1960.

[ref23] Cederbaum L. S.; Chiang Y.-C.; Demekhin P. V.; Moiseyev N. Resonant Auger Decay of Molecules in Intense X-Ray Laser Fields: Light-Induced Strong Nonadiabatic Effects. Phys. Rev. Lett. 2011, 106, 123001 10.1103/PhysRevLett.106.123001. 21517312

[ref24] García-Vela A. Strong Enhancement of the Lifetime of a Resonance State by Using a Combination of Two Laser Pulses. J. Phys. Chem. Lett. 2012, 3, 1941–1945. 10.1021/jz300707g.

[ref25] Calegari F.; Ayuso D.; Trabattoni A.; Belshaw L.; De Camillis S.; Anumula S.; Frassetto F.; Poletto L.; Palacios A.; Decleva P.; Greenwood J. B.; Martín F.; Nisoli M. Ultrafast Electron Dynamics in Phenylalanine Initiated by attosecond Pulses. Science 2014, 346, 336–339. 10.1126/science.1254061. 25324385

[ref26] Ott C.; Kaldun A.; Argenti L.; Raith P.; Meyer K.; Laux M.; Zhang Y.; Blättermann A.; Hagstotz S.; Ding T.; Heck R.; Madroñero J.; Martín F.; Pfeifer T. Reconstruction and Control of a Time-Dependent Two-Electron Wave Packet. Nature 2014, 516, 374–378. 10.1038/nature14026. 25519135

[ref27] Corrales M. E.; González-Vázquez J.; Balerdi G.; Sola I. R.; de Nalda R.; Bañares L. Control of Ultrafast Molecular Photodissociation by Laser-Field-Induced Potentials. Nat. Chem. 2014, 6, 785–790. 10.1038/nchem.2006. 25143213

[ref28] García-Vela A.; Henriksen N. E. Coherent Control of Photofragment Distributions Using Laser Phase Modulation in the Weak-Field Limit. J. Phys. Chem. Lett. 2015, 6, 824–829. 10.1021/acs.jpclett.5b00129. 26262659

[ref29] Corrales M. E.; de Nalda R.; Bañares L. Strong Laser Field Control of Fragment Spatial Distributions from a Photodissociation Reaction. Nat. Commun. 2017, 8, 1345–1351. 10.1038/s41467-017-01139-6. 29116091 PMC5677097

[ref30] García-Vela A. Weak-Field Coherent Control of Molecular Photofragment State Distributions. Phys. Rev. Lett. 2018, 121, 153204 10.1103/PhysRevLett.121.153204. 30362783

[ref31] He L.; Zhang Q.; Lan P.; Cao W.; Zhu X.; Zhai C.; Wang F.; Shi W.; Li M.; Bian X.-B.; Lu P.; Bandrauk A. D. Monitoring Ultrafast Vibrational Dynamics of Isotopic Molecules with Frequency Modulation of High-Order Harmonics. Nat. Commun. 2018, 9, 1108–1114. 10.1038/s41467-018-03568-3. 29549255 PMC5856770

[ref32] Kübel M.; Spanner M.; Dube Z.; Naumov A. Y.; Chelkowski S.; Bandrauk A. D.; Vrakking M. J. J.; Corkum P. B.; Villeneuve D. M.; Staudte A. Probing Multiphoton Light-Induced Molecular Potentials. Nat. Commun. 2020, 11, 2596–2603. 10.1038/s41467-020-16422-2. 32444632 PMC7244592

[ref33] G Arcos C.; García-Vela A.; Sola I. R. Impact of Early Coherences on the Control of Ultrafast Photodissociation Reactions. J. Phys. Chem. Lett. 2024, 15, 1442–1448. 10.1021/acs.jpclett.3c03430. 38291810 PMC10860130

[ref34] Sola I. R.; García-Vela A. Absolute Control over the Quantum Yield of a Photodissociation Reaction Mediated by Nonadiabatic Couplings. Chem. Sci. 2024, 15, 15255–15262. 10.1039/D4SC03235G. 39220160 PMC11350398

[ref35] Dantus M.; Rosker M. J.; Zewail A. H. Real-Time Femtosecond Probing of “Transition States” in Chemical Reactions. J. Chem. Phys. 1987, 87, 2395–2397. 10.1063/1.453122.

[ref36] Zewail A. H. Laser Femtochemistry. Science 1988, 242, 1645–1653. 10.1126/science.242.4886.1645. 17730575

[ref37] Zewail A. H. Femtochemistry Atomic-Scale Dynamics of the Chemical Bond Using Ultrafast Lasers (Nobel Lecture). Angew. Chem., Int. Ed. 2000, 39, 2586–2631. 10.1002/1521-3773(20000804)39:15<2586::aid-anie2586>3.0.co;2-o. 10934390

[ref38] Hentschel M.; Kienberger R.; Spielmann Ch.; Reider G. A.; Milosevic N.; Brabec T.; Corkum P.; Heinzmann U.; Drescher M.; Krausz F. Attosecond Metrology. Nature 2001, 414, 509–513. 10.1038/35107000. 11734845

[ref39] Krausz F.; Ivanov M. Attosecond Physics. Rev. Mod. Phys. 2009, 81, 163–234. 10.1103/RevModPhys.81.163.

[ref40] Balerdi G.; Woodhouse J.; Zanchet A.; de Nalda R.; Senent M. L.; García-Vela A.; Bañares L. Femtosecond Predissociation Dynamics of the Methyl Radical from the 3p_z_ Rydberg State. Phys. Chem. Chem. Phys. 2016, 18, 110–118. 10.1039/C5CP05710H. 26473180

[ref41] García-Vela A. Highly Delocalized Orbiting Resonances. J. Chem. Phys. 2008, 129, 094307 10.1063/1.2974097. 19044870

[ref42] García-Vela A. The Structure of a Resonance State. Chem. Sci. 2017, 8, 4804–4810. 10.1039/C7SC00452D. 28959402 PMC5602369

[ref43] Lovejoy C. M.; Nesbitt D. J. Mode Specific Internal and Direct Rotational Predissociation in HeHF, HeDF, and HeHCl: van der Waals Complexes in the Weak Binding Limit. J. Chem. Phys. 1990, 93, 5387–5407. 10.1063/1.459663.

[ref44] Skodje R. T.; Skouteris D.; Manolopoulos D. E.; Lee S. H.; Dong F.; Liu K. Resonance-Mediated Chemical Reaction: F + HD → HF + D. Phys. Rev. Lett. 2000, 85, 1206–1209. 10.1103/PhysRevLett.85.1206. 10991513

[ref45] Qiu M.; Ren Z.; Che L.; Dai D.; Harich S. A.; Wang X.; Yang X.; Xu C.; Xie D.; Gustafsson M.; Skodje R. T.; Sun Z.; Zhang D. H. Observation of Feshbach Resonances in the F + H2 → HF + H Reaction. Science 2006, 311, 1440–1443. 10.1126/science.1123452. 16527975

[ref46] Che L.; Ren Z.; Wang X.; Dong W.; Dai D.; Wang X.; Zhang D. H.; Yang X.; Sheng L.; Li G.; Werner H. J.; Lique F.; et al. Breakdown of the Born-Oppenheimer Approximation in the F + o-D_2_ → DF + D Reaction. Science 2007, 317, 1061–1064. 10.1126/science.1144984. 17717180

[ref47] Kim J. B.; Weichman M. L.; Sjolander T. F.; Neumark D. M.; Klos J.; Alexander M. H.; Manolopoulos D. E. Spectroscopic Observation of Resonances in the F + H_2_ Reaction. Science 2015, 349, 510–513. 10.1126/science.aac6939. 26228142

[ref48] Shiu W.; Lin J. J.; Liu K. Reactive Resonance in a Polyatomic Reaction. Phys. Rev. Lett. 2004, 92, 103201 10.1103/PhysRevLett.92.103201. 15089205

[ref49] Westermann T.; Kim J. B.; Weichman M. L.; Hock C.; Yacovitch T. I.; Palma J.; Neumark D. M.; Manthe U. Resonances in the Entrance Channel of the Elementary Chemical Reaction of Fluorine and Methane. Angew. Chem., Int. Ed. 2014, 53, 1122–1126. 10.1002/anie.201307822. 24307593

[ref50] Paliwal P.; Deb N.; Reich D. M.; Avoird A. v. d.; Koch C. P.; Narevicius E. Determining the Nature of Quantum Resonances by Probing Elastic and Reactive Scattering in Cold Collisions. Nat. Chem. 2020, 13, 94–98. 10.1038/s41557-020-00578-x. 33257885

[ref51] Chefdeville S.; Stoecklin T.; Bergeat A.; Hickson K. M.; Naulin C.; Costes M. Appearance of Low Energy Resonances in CO-Para-H_2_ Inelastic Collisions. Phys. Rev. Lett. 2012, 109, 023201 10.1103/PhysRevLett.109.023201. 23030157

[ref52] Henson A. B.; Gersten S.; Shagam Y.; Narevicius J.; Narevicius E. Observation of Resonances in Penning Ionization Reactions at sub-Kelvin Temperatures in Merged Beams. Science 2012, 338, 234–238. 10.1126/science.1229141. 23066076

[ref53] Lavert-Ofir E.; Shagam Y.; Henson A. B.; Gersten S.; Klos J.; Żuchowski P. S.; Narevicius J.; Narevicius E. Observation of the Isotope Effect in sub-Kelvin Reactions. Nat. Chem. 2014, 6, 332–335. 10.1038/nchem.1857. 24651201

[ref54] Vogels S. N.; et al. High-Resolution Imaging of Velocity-Controlled Molecular Collisions Using Counterpropagating Beams. Phys. Rev. Lett. 2014, 113, 263202 10.1103/PhysRevLett.113.263202. 25615327

[ref55] Vogels S. N.; Onvlee J.; Chefdeville S.; van der Avoird A.; Groenenboom G. C.; van de Meerakker S. Y. T. Imaging Resonances in Low-Energy NO-He Inelastic Collisions. Science 2015, 350, 787–790. 10.1126/science.aad2356. 26564849

[ref56] Bergeat A.; Onvlee J.; Naulin C.; van der Avoird A.; Costes M. Quantum Dynamical Resonances in Low-Energy CO(j = 0) + He Inelastic Collisions. Nat. Chem. 2015, 7, 349–353. 10.1038/nchem.2204. 25803474

[ref57] Costes M.; Naulin C. Observation of Quantum Dynamical Resonances in Near Cold Inelastic Collisions of astrophysical Molecules. Chem. Sci. 2016, 7, 2462–2469. 10.1039/C5SC04557F. 28660016 PMC5477044

[ref58] Margulis B.; Horn K. P.; Reich D. M.; Upadhyay M.; Kahn N.; Christianen A.; van der Avoird A.; Groenenboom G. C.; Meuwly M.; Koch C. P.; Narevicius E. Tomography of Feshbach Resonance states. Science 2023, 380, 77–81. 10.1126/science.adf9888. 37023184

[ref59] García-Vela A. Active Control of the Lifetime of Excited Resonance States by Means of Laser Pulses. J. Chem. Phys. 2012, 136, 134304 10.1063/1.3698396. 22482549

[ref60] García-Vela A. A Unified Theory of Weak-Field Coherent Control of the Behavior of a Resonance State. Phys. Chem. Chem. Phys. 2019, 21, 7491–7501. 10.1039/C9CP01014A. 30892329

[ref61] García-Vela A. Interference of a Resonance State with Itself: A Route to Control its Dynamical Behaviour. Phys. Chem. Chem. Phys. 2020, 22, 14637–14644. 10.1039/D0CP00392A. 32572415

[ref62] Poullain S. M.; Chicharro D. V.; Zanchet A.; González M. G.; Rubio-Lago L.; Senent M. L.; García-Vela A.; Bañares L. Imaging the Photodissociation Dynamics of the Methyl Radical from the 3s and 3p_z_ Rydberg States. Phys. Chem. Chem. Phys. 2016, 18, 17054–17061. 10.1039/C6CP01558A. 27296907 PMC5049683

[ref63] García-Vela A. Photodissociation of the Methyl Radical: The Role of Nonadiabatic Couplings in Enhancing the Variety of Dissociation Mechanisms. Phys. Chem. Chem. Phys. 2021, 23, 25911–25924. 10.1039/D1CP03293C. 34780593

[ref64] Moiseyev N.; S̆indelka M.; Cederbaum L. S. Laser-Induced Conical Intersections in Molecular Optical Lattices. J. Phys. B:At., Mol. Opt. Phys. 2008, 41, 221001 10.1088/0953-4075/41/22/221001.

[ref65] Demekhin P. V.; Cederbaum L. S. Light-induced conical intersections in polyatomic molecules: General theory, strategies of exploitation, and application. J. Chem. Phys. 2013, 139, 154314 10.1063/1.4826172. 24160520

